# Antioxidant activity and metabolic profile changes in *Poria cocos* fermented by breast-milk-derived *Limosilactobacillus reuteri* HM-R28

**DOI:** 10.3389/fnut.2025.1625875

**Published:** 2025-06-30

**Authors:** Xiaojing Li, Xianping Li, Lu Liu, Junying Zhao, Yue Zhang, Lijun Chen

**Affiliations:** ^1^School of Bioengineering, Dalian Polytechnic University, Dalian, China; ^2^National Engineering Research Center of Dairy Health for Maternal and Child, Beijing Sanyuan Foods Co., Ltd., Beijing, China; ^3^Beijing Engineering Research Center of Dairy, Beijing Technical Innovation Center of Human Milk Research, Beijing Sanyuan Foods Co., Ltd., Beijing, China

**Keywords:** *Limosilactobacillus reuteri* HM-R28, *Poria* polysaccharide, liquid fermentation, antioxidant activity, non-targeted metabolomic

## Abstract

**Introduction:**

The fermentation of lactic acid bacteria can alter the nutraceutical and functional efficacy of food and medicinal products, particularly when involving bioactive compounds similar to those in *Poria cocos* (*P. cocos*), which include polysaccharides and triterpenoids. In this study, we used *Limosilactobacillus reuteri* HM-R28 (*L. reuteri* HM-R28) from breast milk to ferment *P. cocos* in a liquid state and investigated changes in the physicochemical properties and metabolic composition before and after fermentation.

**Methods:**

Additionally, we evaluated the effect of *L. reuteri* HM-R28 on the antioxidant activity and polysaccharide content of the *P. cocos* fermentation broth to elucidate its role.

**Results and discussion:**

Fermentation with *L. reuteri* HM-R28 enhanced the antioxidant activity of the fermentation solution. Liquid fermentation significantly altered the metabolites of *P. cocos*. Based on the broadly targeted metabolomics analysis, a total of 549 differential metabolites were detected in the fermentation broth before and after fermentation, with 254 metabolites significantly upregulated and 295 metabolites significantly downregulated. These differential metabolites were associated with 41 metabolic pathways, primarily involving lipids and lipid constituents, organic acids and their derivatives, and heterocyclic organic compounds. Liquid fermentation also increased antioxidant compounds such as sedanolide, pomegranate acid, and epigallocatechin, along with aromatic volatile substances such as delta-tridecanolactone, *γ*-nonanolactone, and 3-acetyl-2, 5-dimethylfuran. These insights establish a conceptual framework for advancing the use of human milk-derived lactic acid bacteria for fermenting medicinal and edible homologous products. To our knowledge, this is the first study to elucidate the role of human milk-derived probiotics in enhancing the bioactive compounds of *P. cocos* through fermentation. Our findings can provide a theoretical foundation for the development of medicinal and edible homologous products fermented with human milk-derived *Lactobacillus* strains.

## 1 Introduction

*Poria cocos* (*P. cocos*), a dried mycelium of the fungus *P. cocos* (Polyporaceae), is native to China and has been used for over 2,000 years in traditional medicine across China, Japan, South Korea, and North America (1). It is rich in polysaccharides and triterpenoids, which contribute to its wide range of pharmacological activities, including anti-inflammatory, anticancer, immunomodulatory, antioxidant, gut microbiota-regulating, and calming effects on the nervous system (2). Recorded in Mhennong Materia Medica, it is associated with the heart and spleen meridians (3). *P. cocos* has been utilized in traditional Chinese medicine for over 2,000 years for various therapeutic and health purposes (4). According to the principle of “food and medicinal homology,” it qualifies as both a functional food and a medicinal substance due to its long history of safe use, nutritional value, and health benefits (5). Owing to its diverse pharmacological properties, *P. cocos* has been widely used in traditional Asian medicine and is recognized as a safe functional food due to its long-term consumption history, nutritional richness, and substantial health benefits (6). However, challenges in the effective utilization of its bioactive components remain (7).

Liquid fermentation technology, originally developed for antibiotic production, has been adapted for *P. cocos* cultivation by incorporating medicinal and food-homologous substrates. This method enhances the acid content in *P. cocos* mycelium, improves nutritional and medicinal value, reduces costs, and decreases dependency on wild pine-based cultivation (8). Moreover, it enhances flavor and bioavailability, supporting large-scale industrial development of *P. cocos* products.

Breast milk is a complex and highly variable biological fluid that contains numerous proteins, lipids, carbohydrates, several bioactive compounds, as well as probiotics. Extensive studies have shown that probiotics derived from breast milk play a key role in regulating gut microbiota, alleviating constipation, and treating conditions such as mastitis (9). Being naturally sourced, these probiotics provide scientific evidence to support the development of specialized infant formulas and other functional fermented products.

To the best of our knowledge, no previous study has employed breast milk-derived probiotics fermentation to change the composition of *P. cocos* active ingredients, increase biological activity, or enrich taste and flavor. In this study, we aimed to investigate the liquid fermentation of *P. cocos* with breast-milk-derived *Limosilactobacillus reuteri* HM-R28 (*L. reuteri* HM-R28). By increasing the hydrosoluble polysaccharide content in the *P. cocos* homogenate, its effect on promoting the growth of *L. reuteri* HM-R28 was observed. Additionally, we analyzed the physicochemical properties of the fermentation broth, along with its antioxidant activity. Using non-targeted metabolomics technology, we investigated the dynamic changes in metabolites within the fermentation broth after fermenting *P. cocos* with *L. reuteri* HM-R28. Given that research on *P. cocos* fermentation using lactic acid bacteria remains limited, this study explores how *P. cocos* influences lactic acid bacteria proliferation and the bioactivity of fermentation products, aiming to promote research on lactic acid bacteria from human sources, and provide new ideas for developing *P. cocos* functional products.

## 2 Materials and methods

### 2.1 Materials and reagents

Poria powder, *P. cocos* polysaccharides (120 mesh, Lot No. SNT231105) was purchased from Fufeng Snout Biotechnology Co. MRS broth culture medium was purchased from Beijing Landbridge Technology Co., LTD. Sterile PBS (Cell-specific, 0.1 M, pH 7.2–7.4) was sourced from FEIMOBIO. The free radical reagent DPPH (1,1-diphenyl-2-trinitrophenylhydrazine) was sourced from Tokyo Chemical Industry Co., Ltd. (TCI). Phenanthroline, triphenylphenol, and glucose standards were purchased from Sigma. Hydrogen peroxide, hydrochloric acid, potassium ferrocyanide, and trichloroacetic acid were acquired from National Group Chemical Reagent Co., Ltd. Tris–HCl was obtained from Thermo Fisher, while ferrous sulfate was purchased from Xi’an Chemical Reagent Factory. Both sulfuric acid and phenol are of analytical reagent grade, and they were acquired from Beijing Chemical Works and Tianjin Xinyuan Chemical Co., Ltd., respectively.

### 2.2 Strain and culture conditions

*L. reuteri* HM-R28 (strain preservation number: CGMCC No. 23658) was isolated from the breast milk of healthy Chinese women. Its probiotic characteristics have been comprehensively evaluated by our research group. The strain was selected for this study based on its strong synergistic performance in co-fermentation with *P. cocos*. It is currently preserved at the National Maternal and Infant Dairy Health Engineering Technology Research Center. The strain was subcultured twice in MRS broth medium at 37°C shaking at 180 rpm. The bacterial solution was diluted with sterile PBS to a concentration of 0.500 ± 0.02 MCF/L for subsequent experiments.

### 2.3 *Poria* liquid fermentation

*P. cocos* powder (25% w/w) was weighed, added to distilled water, and soaked for 12 h. This concentration corresponds to the 1:3 solid–liquid ratio for polysaccharide extraction, as determined by orthogonal optimization experiments. Subsequently, 5% cellulase was added, and the mixture was placed in a 70°C water bath for 1 h before it was set aside for later use (Cellulase was heat-inactivated prior to fermentation). Following sterilization of the MRS liquid medium at 121°C for 20 min, it was inoculated with pretreated Poria powder and diluted *L. reuteri* HM-R28. Thereafter, it was incubated for 24 h in a shaker incubator at 37°C and 180 rpm. This time point was selected based on preliminary time-course assays showing maximal microbial proliferation without diminishing returns. The blank control contained MRS liquid medium inoculated with the *L. reuteri* HM-R28 strain but without *Poria*. The experiment was conducted in triplicate with three parallel sets per group.

### 2.4 Enumeration of viable bacteria

Viable bacteria in fermentation samples were quantified using the dilution gradient microplate counting method. Sample (1 mL) was incorporated to 9 mL of sterilized PBS, mixed thoroughly, and serially diluted 10-fold. Using a sterile pipette, the bacterial solution was diluted at gradients of 10^−5^, 10^−6^, and 10^−7^, respectively, and inoculated onto plates corresponding to each dilution. The plates were incubated invertedly at 37°C for 24–48 h, and viable bacteria counts were recorded. N1 represents the viable bacteria count in the *P. cocos* fermentation group, while N0 indicates the viable bacteria count in the single-strain group. The bacterial proliferation rate was calculated using the following equation (1):


(1) $$\text{Growth rate}(\%)=\frac{N1-N0}{N0}\times 100$$

### 2.5 Determination of water-soluble polysaccharide content

#### 2.5.1 Preparation of glucose standard curve

The concentration of hydrosoluble polysaccharide was measured using the sulfuric acid-phenol method with slight modifications (10). Briefly, a 0.1 mg/mL glucose standard solution was prepared from dried, constant-weight glucose and adjusted with distilled water to concentrations of 0, 0.01, 0.02, 0.04, 0.06, 0.08, and 0.1 mg/mL. For each standard concentration, 200 μL of mixed with an equivalent volume of a 5% phenol solution. Immediately thereafter, 1 mL of concentrated sulfuric acid was added rapidly, followed by vigorous shaking to ensure complete reaction. The mixture was then left undisturbed for 10 min. After thorough mixings, it was incubated in a 40°C thermostatic bath for 15 min. The 490 nm absorbance value was recorded spectrophotometrically.

#### 2.5.2 Determination of extracellular water-soluble polysaccharides

*P. cocos* powder was autoclaved with distilled water at 121°C for 20 min, equilibrated to ambient temperature and then subjected to liquid-state fermentation with *L. reuteri* HM-R28. Samples collected before and after fermentation centrifuged at 6,000 rpm for 10 min. The resulting supernatant was used to measure the OD value at 490 nm. The water-soluble polysaccharide concentration was determined using a glucose calibration curve.

### 2.6 Physical and chemical analysis

The growth curve of *L. reuteri* HM-R28 in the fermentation broth was determined d at 600 nm using a UV spectrophotometer. The pH was determined using a digital pH meter (FE28, Mettler Toledo (Shanghai) Co., Ltd.). The total sugar content, using glucose as the standard, was analyzed using the Dubois method at 490 nm, and reducing sugars were quantified using the DNS colorimetric assay at 540 nm (11).

### 2.7 Determination of total phenolic and total flavonoid content

The overall phenolic concentration was measured using a modified Folin–Ciocalteu method (12). A mixture of 1.0 mL Folin–Ciocalteu reagent and 2 mL of the sample supernatant was incubated reacted at ambient temperature for 10 min. Subsequently 3 mL of 7.5% Na_2_CO_3_ solution was added, subsequently incubated in a 45°C water bath for 1.5 h. Absorbance was quantified at 765 nm. Gallic acid served as the standard. The standard curve equation was as follows: y = 0.1508x + 0.0876, with *R*^2^ = 0.9924.

Total flavonoid content was quantified using a modified sodium nitrite- aluminum nitrate spectrophotometric (13). A 2 mL aliquot of the fermentation supernatant was blended with 0.5 mL of 5% NaNO_2_ and maintained at ambient temperature for 6 min. Thereafter, 0.5 mL of 10% Al (NO_3_)_3_ was added, followed by additional incubation of 6 min. Next, 4 mL of 4% NaOH and 3 mL of 60% ethanol were added, mixed thoroughly, and allowed to settle for 15 min. Finally, the absorbance was recorded at 510 nm with a UV–Vis spectrophotometer. Rutin was used as a standard, with the standard curve equation presented as y = 0.1436 + 0.0621, *R*^2^ = 0.9922.

### 2.8 Determination of antioxidant activity

#### 2.8.1 Determination of *DPPH* free radical scavenging ability

The DPPH radical elimination assay used in this study was partially modified based on an earlier approach (14). A 1 mL sample was thoroughly blended with 1 mL of 0.2 mmol/L anhydrous ethanol-DPPH and incubated under dark conditions at room temperature for 30 min. The mixture was centrifuged at 6,000 rpm for 10 min, and the optical density of the supernatant (A_i_) was determined at 517 nm (A_i_). Subsequently, scavenging efficiency toward DPPH radicals was assessed using a computational model equation (2):


(2) $$\text{DPPH radical scavenging}\;(\%)=(1-\frac{\mathrm{Ai}-\mathrm{Aj}}{A0})\times 100\;\;$$

Here, A_i_ denotes the optical density of the sample blended with DPPH. A_0_ is an equal volume of anhydrous ethanol solution, and A_j_ denote a sample mixed with anhydrous ethanol-DPPH.

#### 2.8.2 Hydroxyl radical suppression capacity measurement

Hydroxyl radical antioxidant efficacy assay was used in the present study, with few modifications (15). Briefly, sample (1 mL) was mixed with 1 mL of 2.5 mmol/L O- phenanthroline, 1 mL of PBS (pH = 4), and 1 mL of 2.5 mmol/L FeSO_4_. Thereafter, 1 mL of 20 mmol/L H_2_O_2_ was supplemented, and the mixture was thoroughly mixed. After incubation in a 37°C hydrothermal bath for 1.5 h, the absorbance (A_i_) was determined at 536 nm. Hydroxyl radical antioxidant efficacy capacity was accumulated using the succeeding equation (3):


(3) $$\begin{array}{c} \text{Hydroxyl radical scavenging}(\%)=\frac{\mathrm{Ai}-\mathrm{Aj}}{A0-\mathrm{Aj}}\times 100\;\;\; \end{array}$$

Here, A_j_ was obtained by replacing 1 mL of the sample with an equivalent amount of deionized water, while A_0_ was obtained by replacing 1 mL of H_2_O_2_ with 1 mL of aqueous distillate.

#### 2.8.3 Assessment of superoxide anion radical neutralizing ability

The scavenging of superoxide anion radical assay was modified from the previous method (16). Briefly, 1 mL of sample was mixed with 3 mL of 0.05 mol/L Tris–HCl solution and allowed to stand at 25°C for 20 min. Subsequently, 0.4 mL of 25 mmol/L catechol was introduced, and the mixture was incubated at room temperature for 4 min. The reaction was stopped by introducing 0.5 mL of 8 mol/L HCl, and the absorbance (A_i_) was measured at 320 nm. The removal of superoxide anion radical capacity was determined using the subsequent equation (4):


(4) $$\begin{array}{c} \begin{array}{l} \text{Superoxide anion radical scavenging ability}(\%)\\ =(1-\frac{\mathrm{Ai}-\mathrm{Aj}}{A0})\times 100\;\;\; \end{array} \end{array}$$

Here, A_0_ represents the absorbance when pyrogallol is substituted with an identical volume of distilled water, while A_j_ represents an identical volume of purified water.

### 2.9 Non-targeted metabolomics analysis

#### 2.9.1 Metabolite extraction

A 1 mL specimen was freeze dried in an Eppendorf tube, resuspended in 100 μL of precooled 80% methanol, and centrifuged in an ice bath under centrifugation at 15,000 g for 15 min at 4°C. The supernatant, containing 80% methanol, was then adjusted with LC–MS grade water to achieve an ultimate concentration of 53% methanol. Subsequently, the supernatant was centrifuged again, collected and analyzed using an LC–MS/MS system. For quality control (QC) specimens, an equal volume of each experimental specimen was combined to create pooled QC specimens. A control blank was prepared by replacing the sample treated with a 53% aqueous solution of methanol and subjecting it to the same pretreatment process. The *L. reuteri* HM–R28 fermentation group was designated as group B, while the *L. reuteri* HM-R28 fermented Poria group was designated as group PB. Each group contained six parallel samples.

#### 2.9.2 *UHPLC–MS/MS* detection and analysis

Off-target metabolite analysis was performed using a Vanquish ultra-high-performance liquid chromatography (UHPLC) system integrated with an Orbitrap Q Exactive™ HF/Q Exactive™ HF–X mass spectrometer. In the positive mode, the eluent consisted of eluents A (0.1% formic acid aqueous solution) and B (methanol) pumped at a volumetric rate of 0.2 mL/min. In the negative mode, mobile phase A comprised an ammonium acetate solution (5 mM, pH-adjusted to 9.0), while mobile phase B was methanol. Linear gradient elution was conducted using a pump that delivered a volumetric rate of 0.30 mL/min as follows: 2% B at 1.5 min, increasing to 85% B at 3 min, reaching 100%B at 10 min, returning to 2% B at 10.1 min, and maintaining 2% B until 12 min. The ESI source was operated at an electrospray voltage of 3.5 kV, a coaxial gas flux of 35 psi, and an auxiliary gas flow rate of 10 L/min. The ion transfer capillary temperature was maintained at 320°C, while the ion introduction radiofrequency level was maintained at 60 and an auxiliary gas thermal module regulated to 350°C (17, 18).

#### 2.9.3 Data processing

Non-targeted metabolomics multivariate approaches were employed to examine the effects of *L. reuteri* HM–R28 fermentation on the metabolic profile of the *P. cocos* fermentation broth. Following UPLC–MS analysis, untreated mass spectrometry datasets underwent computational preprocessing through Compound Discoverer 3.3 for peak identification and alignment. The characterized molecular features were algorithmically aligned with three orthogonal spectral libraries to enable compound annotation and semi-quantitative metabolic profiling.

### 2.10 Statistical analyses

The metaX computational pipeline (v8.2) executed supervised learning protocols through PLS-DA algorithms coupled with unsupervised PCA clustering for metabolic pattern differentiation. The t-test was applied to establish statistical significance. The fold-change (FC) was calculated based on the quantitative profiles of all experimental replicates for each metabolite in the compared cohorts. Differentially abundant metabolites were identified based on a variable importance in the projection (VIP) score > 1.0, an FC threshold of > 1.2 or FC < 0.833, and significance was defined as *p* < 0.05 (13). The functional and metabolic pathways of these metabolites were examined using the KEGG database. For the untargeted metabolomics analysis, the false discovery rate (FDR) correction was applied to six independent experiments, retaining only differential metabolites with a q-value < 0.05. Other experimental analyses were conducted with three independent replicates. Basic data were organized using Microsoft Excel 2010, and data analysis and visualization were performed Graph Pad Prism software. Experimental outcomes are reported as mean ± standard deviation (SD). A two-tailed independent t-test was conducted for pairwise comparisons, whereas analysis of variance (ANOVA) was applied to analyze differences across multiple groups.

## 3 Results and discussion

### 3.1 *Poria cocos* polysaccharide promotes proliferation of lactic acid bacteria

To investigate the relationship between *P. cocos* polysaccharides and *L. reuteri* HM–R28, we plotted glucose content on the x-axis and optical density (OD) values on the y-axis. The resulting linear regression relationship, Y = 6.2383X + 0.0789 (*R*^2^ = 0.999), was used for the determination of polysaccharide content in subsequent experiments.

**Figure 1:**
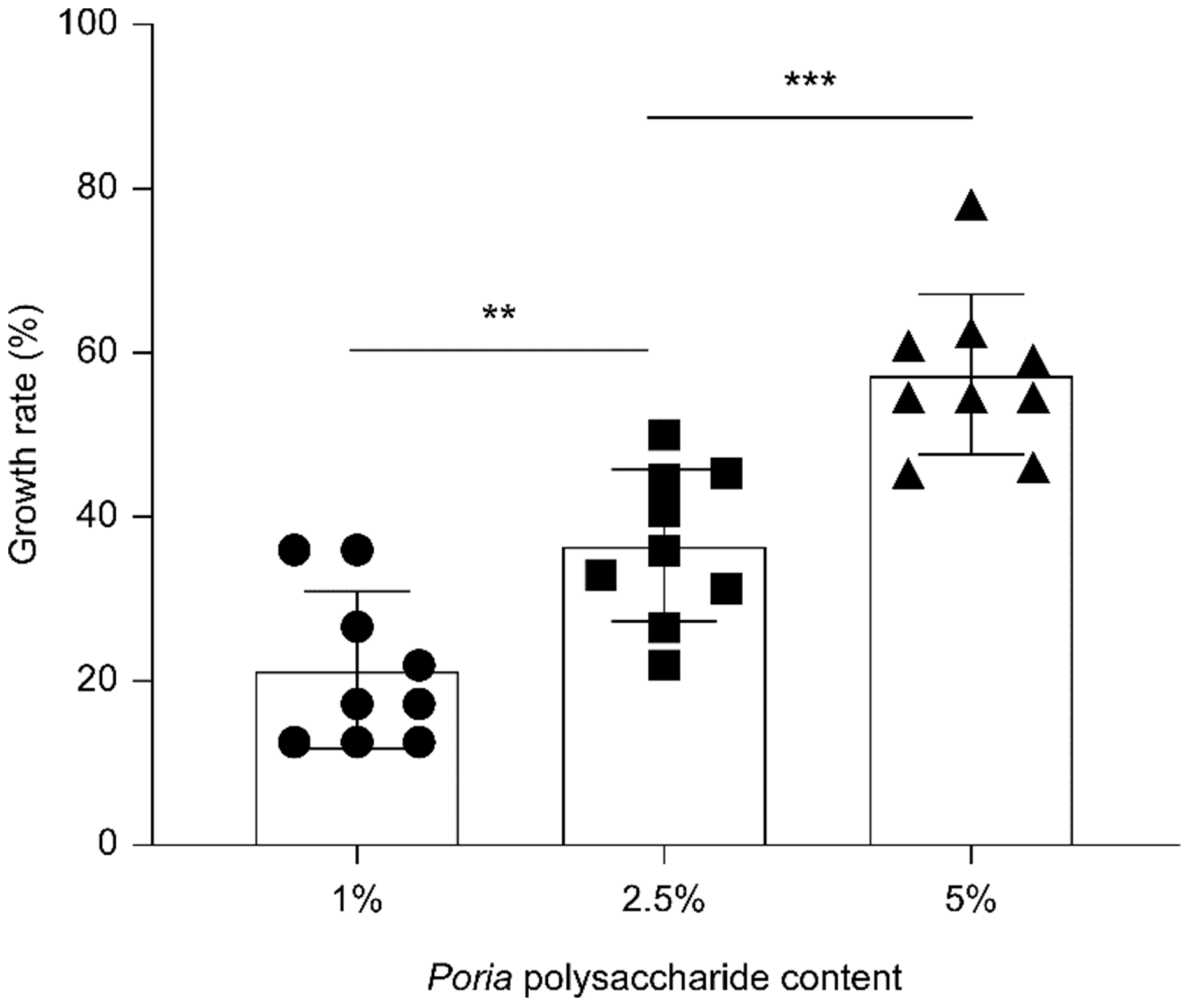
*Poria cocos* polysaccharides promote strain *L. reuteri* HM-R28 proliferation. * indicates *p* < 0.01, *** indicates *p* < 0.001.

The proliferation of *L. reuteri* HM–R28 derived from breast milk, varied with the addition of different concentrations of *P. cocos* powder polysaccharides dissolved in MRS broth medium. At concentrations of 1, 2.5, and 5% *P. cocos* polysaccharide (Figure 1), the proliferation rates of strain *L. reuteri* HM-R28 were 21.37, 35.96 and 54.71%, respectively, indicating a positive correlation between the *P. cocos* polysaccharide concentration and the proliferation rate of *L. reuteri* HM-R28. The water-soluble polysaccharides likely served as a key carbon and energy source for *L. reuteri* HM-R28, as indicated by the significantly higher viable bacterial count observed in the experimental group compared to the control. This finding is consistent with previous studies demonstrating that exogenous polysaccharides can promote the proliferation of lactic acid bacteria (LAB) by providing fermentable substrates and modulating metabolic activity (19). Similarly, supplementing Roy’s mucopramium culture medium with coix seed polysaccharides resulted in a viable bacterial count of 12.24 log10 CFU/mL, demonstrating a significant statistical advantage over the control group (9.16 log10 CFU/ml, *p* < 0.05) (20). A similar phenomenon has been previously reported in the bergamot juice fermentation by *L. plantarum*, in which sugar serves as a carbon source and an energy source for bacterial growth (21). These findings suggest that *P. cocos* polysaccharides may enhance biomass accumulation in *L. reuteri* HM-R28 by activating glycolytic pathways or inducing extracellular enzyme production.

### 3.2 Changes in physical and chemical properties during fermentation

**Figure 2:**
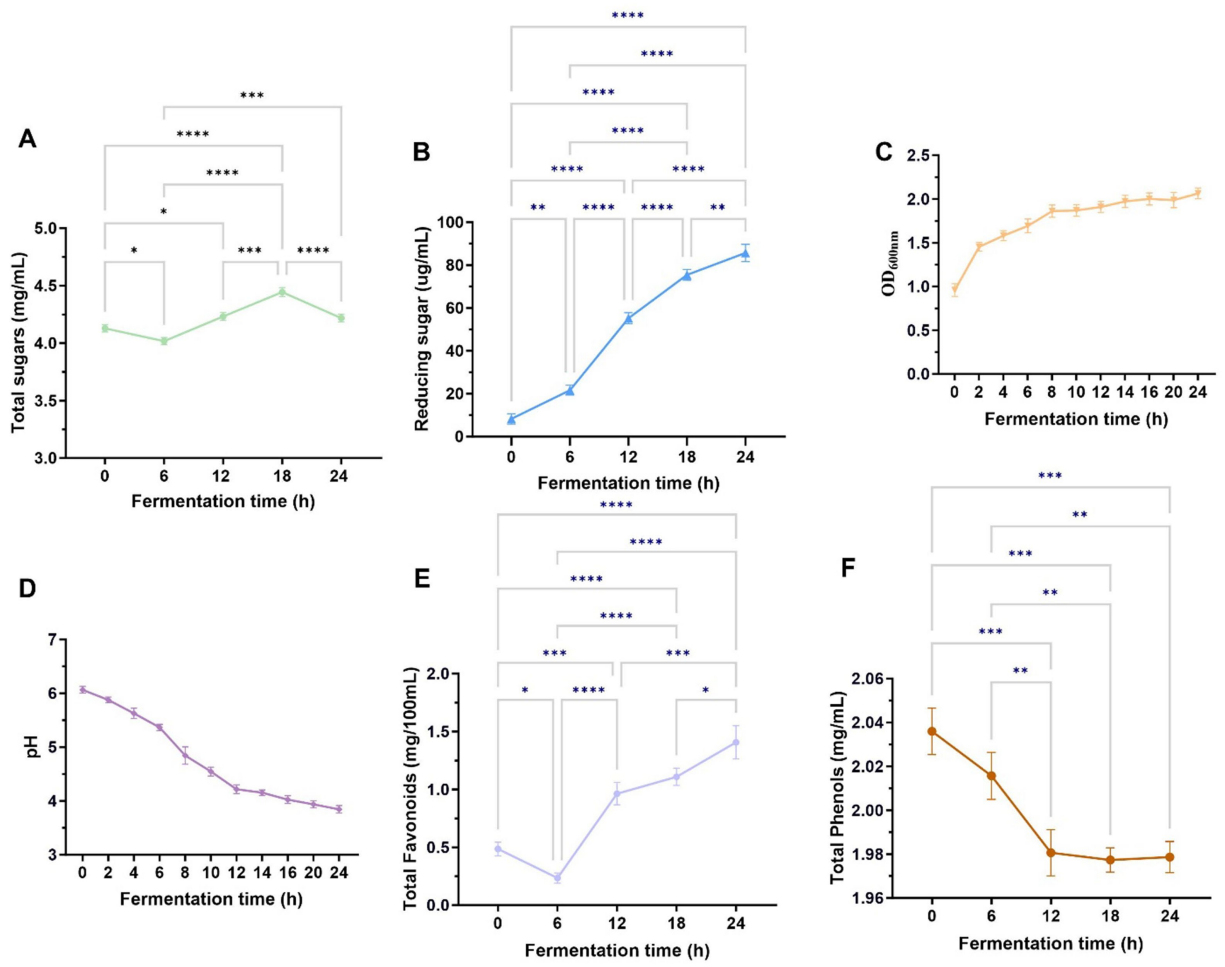
Growth curves of total sugars **(A)**, reducing sugars **(B)**, *L. reuteri* HM-R28 **(C)**, pH **(D)**, total flavonoid content **(E)**, and total phenol content **(F)** in the fermentation solution differed during the fermentation process. * indicates *p* < 0.01, *** indicates *p* < 0.001.

Figures 2A,B show the changes within the total sugar and reduced sugar matrix in fermentation broth over different fermentation periods. The total sugar content demonstrated an initial reduction, later increasing, and then another decline. However, as seen in Figure 2A, the total sugar content showed a general upward trend increasing. We hypothesized that in the initial stage of fermentation, *L. reuteri* HM-R28 would metabolize fermentable sugars present in *P. cocos*, leading to a reduction in the total sugar content (11). At 18–24, the growth of *L. reuteri* HM-R28 may have been restricted by the accumulation of high concentrations or other inhibitory factors, causing, a subsequent decline in total sugar content. We further hypothesized that the overall increase within the total sugar during fermentation was due to the production of lactic acid by *L. reuteri* HM-R28. This acidification could have facilitated the breakdown of *P. cocos* components, releasing additional sugars under specific conditions (22). The continuous increase in reduced sugar content, as seen in Figure 2B, may be attributed to the enzymatic hydrolysis of polysaccharides into smaller sugar molecules during fermentation, thereby increasing the concentration of reducing sugars in the fermentation broth.

The survival of lactic acid bacteria is a crucial prerequisite for their probiotic functionality. Figure 2C shows that the growth rate of *L. reuteri* HM-R28 in the fermentation broth was the highest within the first 0–6 h of fermentation, likely due to the initial were abundance of nutrients. By the 12 h mark, the growth rate of *L. reuteri* HM-R28 began plateau. We speculate that *L. reuteri* HM-R28 underwent rapid proliferation throughout the fermentation process, and the continuous nature of its growth curve reflects a stable and healthy fermentation state (11). The pH value of the biotransformation broth exhibited overall downward trend as fermentation progressed (Figure 2D). This decline may be attributed to the metabolic characteristics of *L. reuteri* HM-R28. In the early stages of fermentation, *L. reuteri* HM-R28 produces lactic acid as it ferments total sugar (23). Additionally, throughout the fermentation process, lysine in the broth was degraded, and alkaline compounds such as histidine, were metabolized. This led to a continuous decrease in pH, further indicating active microbial fermentation and organic production.

### 3.3 Changes in total phenolic and flavonoid content during fermentation process

Foods rich in total phenols and flavonoids positively influence multiple physiological functions in humans, thereby promoting overall health (24). The total phenol and flavonoid content in fruits and vegetables exist in both bound and free states. During fermentation, lactic acid bacteria (LAB) hydrolyze complex molecules into free or simpler forms, enhancing the bioavailability of phenol and flavonoids. Different strains of lactic acid bacteria secrete distinct enzymes during fermentation, each exerting unique effects on phenols and flavonoids (25). The total flavonoid levels decreased during the first 0-6 h of fermentation, rapidly, increased at 6–12 h, and showed a slight increase 12–24 h, ultimately demonstrating an overall upward trend throughout the entire fermentation process (Figure 2E). The initial decline in flavonoid levels observed during the first 0–6 h is likely attributable to microbial utilization of flavonoids as a carbon source or the enzymatic hydrolysis of glycosylated flavonoids into aglycones, which may not be fully detected by total flavonoid assays (26). In contrast, the subsequent increase between 6 and 24 h likely reflects microbial biotransformation processes, including the deglycosylation of flavonoid precursors by bacterial *β*-glucosidases or the action of polyphenol oxidases or both. These enzymatic activities may produce bioactive derivatives such as smaller phenolic compounds or quinones, which could have contributed to the observed rise in total flavonoid content (26, 27). Additionally, co-fermentation with Monascus Anka and *Saccharomyces cerevisiae* enhances flavonoid content in citrus peel, thereby improving its characteristic compounds ingredients and overall quality. Flavonoid compounds may be utilized by microorganisms as nutrients or transformed into other bioactive compounds through enzyme, leading to a reduction in their content (13, 28). We speculated that during the initial 0–6 h (Figure 2F), *L. reuteri* HM-R28 in the fermentation broth utilized certain phenolic precursor compounds present in *P. cocos* itself to convert them into phenolic compounds through enzymatic reactions (29). After 6 h, the phenol-type compounds formed in the fermentation solution may undergo further conversion into quinone compounds or participate in polymerization reactions, producing more complex polymers. These complex compounds may not be detected as total phenols, leading to an apparent decline in the measured phenolic content (25). This hypothesis was confirmed when we noted an increase in levels of lipids, polyketides, and organic heterocyclic compounds in the fermentation broth after fermentation. These findings support our hypothesis, although the effects of *L. reuteri* HM-R28 and *P. cocos* fermentation on total flavonoids and total phenolic compounds require further investigation.

### 3.4 Microbial metabolism-mediated antioxidant activity transitions

**Figure 3:**
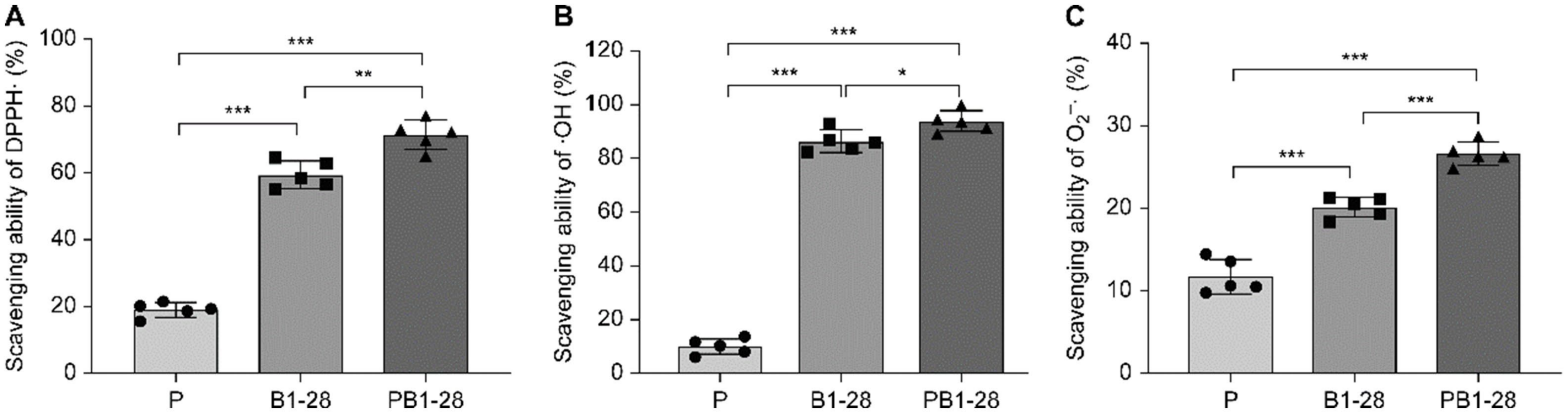
Changes in antioxidant activity during fermentation. **(A)** DPPH clearance capacity, **(B)** OH scavenging capacity, and **(C)** O_2_^−^ scavenging capacity. *** represents *p* < 0.001, P represents an unblocked sample of Poria on the liquid, *L. reuteri* HM-R28 represents *L. reuteri* HM-R28 bacterial liquid sample, PHM-R28 represents Poria and strain *L. reuteri* HM-R28 cleaning samples on the same fermentation sample.

Lactic acid bacteria fermentation generalizability in enhancing the antioxidant activity of medicinal and edible homology (30, 31). To evaluate the antioxidant activity of *P. cocos* fermented by *L. reuteri* HM-R28, we measured its DPPH, hydroxyl, and superoxide anion radical scavenging activities (Figures 3A–C). The removal rates of these radicals followed a similar trend across the different groups, with the fermented *P. cocos* exhibiting higher scavenging activity than non-fermented *P. cocos* and the *L. reuteri* HM-R28 solution. The scavenging activities of DPPH, hydroxyl, and superoxide radicals of fermented *P. cocos* solution were higher than those of the *P. cocos* solution alone by 3.77, 9.34, and 2.77 times, respectively; these values were also higher than those observed for the *L. reuteri* HM-R28 strain by 1.20, 1.09 and 1.32 times, respectively. Our findings align with those of a previous study, which demonstrated that the radical-neutralizing capacity of *P. cocos* improves significantly after fermentation (21). Polysaccharides are considered key contributors to the antioxidant properties of *P. cocos*. During fermentation, *Lactobacillus* releases *β*-glucosidase, an enzyme that degrades polysaccharides and may release their bioactive components (32). Generally, *L. reuteri* HM-R28 fermentation significantly improved both the antioxidant capacity and nutritional value of *P. cocos*, although its underlying antioxidant mechanisms require further investigation.

### 3.5 Metabolic profile analysis before and after fermentation

**Figure 4:**
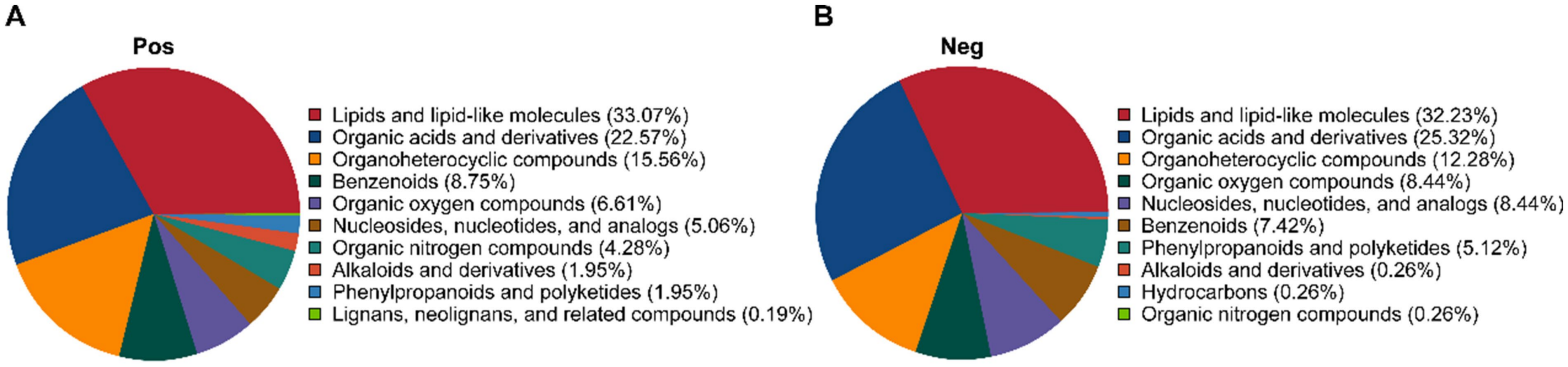
Metabolic category cake-shaped diagram. **(A)** Positive and **(B)** negative ion modes.

The dynamic changes in metabolites during *P. cocos* and *L. reuteri* HM-R28 co-fermentation, as well as *L. reuteri* HM-R28 single-strain fermentation were analyzed using non-targeted metabolomics technology. In the positive ion mode, 890 metabolites were identified (Figure 4A), including 33.07% of lipid and lipid analogs, 22.57% of organic acids and their compounds, 15.56% of organic heterocycles, and 8.75% of benzene compounds. In the negative ion mode, 490 metabolites were identified (Figure 4B), including 32.23% lipid and lipid analogs, 25.32% organic acids and their compounds, 12.28% organic heterocycles, 8.44% organic compounds with oxygen, and 8.44% nucleosides, nucleotides, and related compounds.

### 3.6 Differential metabolite analysis

**Figure 5:**
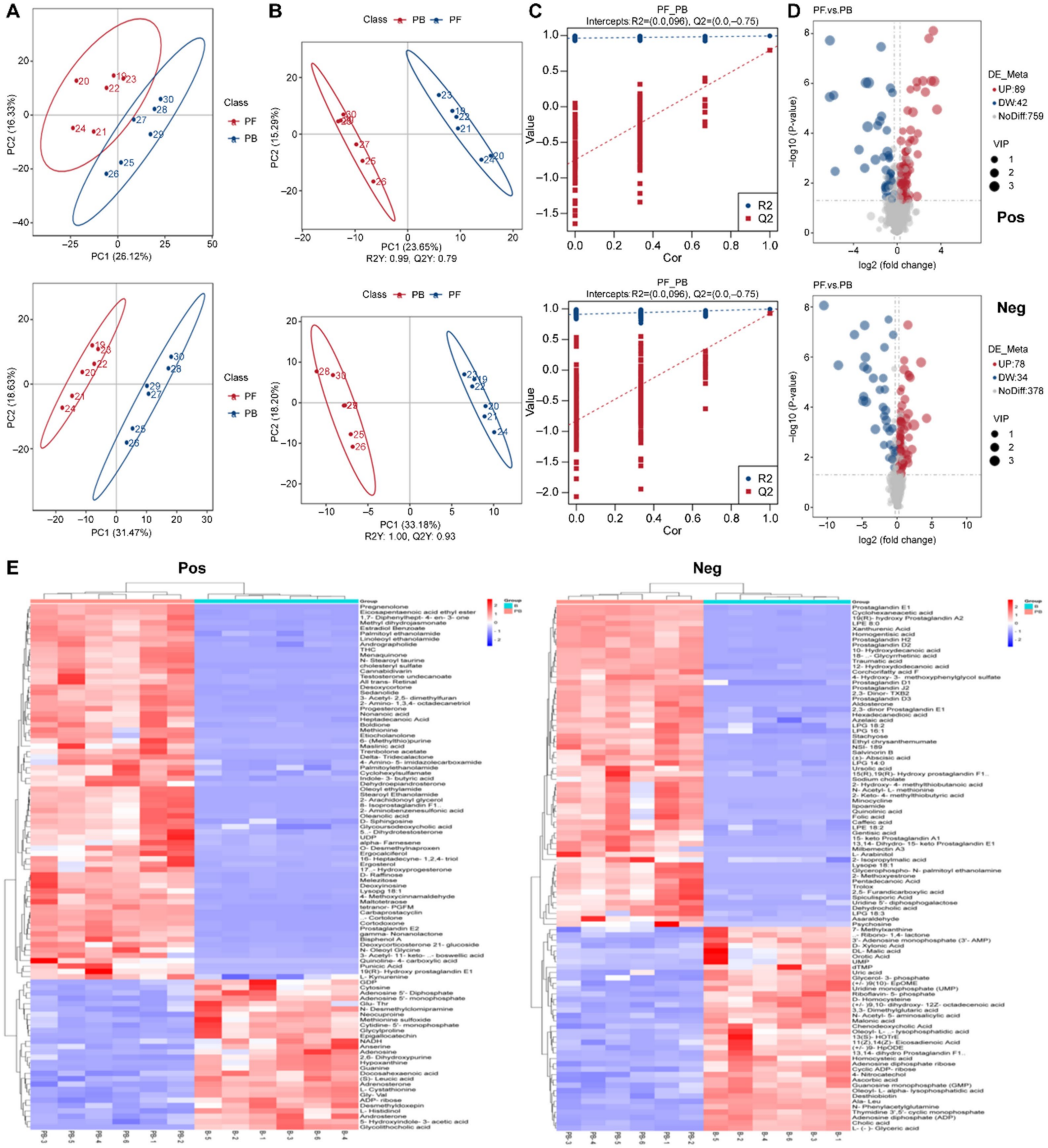
Composition analysis of the fermentation broth. **(A)** PCA, **(B)** orthogonal PLS-DA, and **(C)** overfitting verification in the positive ion mode. **(D)** Volcano plot of differential metabolites. **(E)** Hierarchical cluster heatmap of metabolites. PCA, principal component analysis; PLS-DA, partial least squares discriminant analysis.

Figure 5A illustrates a significant classification effect between samples from the *L. reuteri* HM-R28 fermentation (group B) and *L. reuteri* HM-R28 fermented-*P. cocos* group (group PB) groups. Samples within each group clustered closely, indicating the co-fermentation of *P. cocos* and lactic acid bacteria. Unlike single fermentation metabolites of lactic acid bacteria, the total contribution rates in positive and negative ion settings reached 57.82 and 64.91%, respectively. Additionally, the Pearson correlation coefficient for QC samples was determined using the relative quantification values of metabolites (11). In the present study, the QC samples were highly concentrated in both modes (*R*^2^ > 0.98), confirming the robustness of the detection process and superior data quality. Therefore, the metabolic group was suitable for biological analysis.

Orthogonal partial least squares discriminant analysis (OPLS-DA) eliminates unrelated variables, enhancing to improve model efficiency and facilitating the identification of reliable differential metabolites (11). Herein, the positive and negative ion modes reached 55.54 and 62.42%, respectively, with pronounced inter-group differences (Figure 5B) and an *R*^2^ of 1.00 and Q^2^ > 0.98, confirming the stability and reliability of the model (33). Model accuracy was evaluated using the parameters R^2^X, R^2^Y, and Q^2^. R^2^X indicates the ability of the model to explain changes in the X variable, R^2^Y represents the fit of the model to the Y variable, and Q^2^ reflects the model’s ability to predict outcomes. The R^2^X, R^2^Y, and Q^2^ values range between 0 and 1, with values close to 1 indicating at better model fit. A model is considered stable and interpretable when *R*^2^ exceeds Q^2^, and Q^2^ remains above 0, confirming the absence of overfitting (Figure 5C). Therefore, the OPLS-DA model was deemed suitable for identifying differentiated metabolites pre- and post-fermentation.

Multivariate statistical analysis was conducted using the obtained VIP of the OPLS-DA model, applying a threshold of VIP > 1.0, FC > 1.2 or FC < 0.833 and *p* < 0.05. To better visualize the changes in the differential metabolites, volcano plots were generated (Figure 5D). In the positive ion mode, 348 metabolites were annotated, with 156 up-regulated and 192 down-regulated. In the negative ion mode, 201 metabolites were annotated, with 98 up-regulated and 103 down-regulated. These findings indicate significant differences in the metabolic composition of the fermentation broth before and after fermentation. Based on this, a clustered heat map analysis of the differential metabolites was performed (Figure 5E), which was dominated by lipid analog, organic acid derivatives, and organic heterocyclic compounds in the positive ion mode. Specifically 89 lipid analog, 46 organic acid derivatives, and 30 organic heterocycles were identified. Additionally, 10 organic oxygen compounds, 11 organic nitrogen compounds, 15 benzene ring-type compounds, 13 nucleotide-type differential metabolites, and 5 phenylpropanoids and polyketides were detected. Lipids are generally challenging for microorganisms to metabolize during fermentation, and their content usually increases over time (34). *L. buchneri* uses xylose as a carbon source to produce short-chain fatty acids under anaerobic conditions, facilitating its growth and lipid accumulation (35). Oleanolic acid, the primary triterpenoid in *P. cocos*, showed a significant increase in the fermentation broth (*p* = 8.2278E-06, VIP = 1.511939271) after co-fermentation with *L. reuteri* HM-R28 compared with that in the reference group. The findings indicate that *L. reuteri* HM-R28 fermentation from breast milk could improve the medicinal and health benefits of *P. cocos*. However, the specific mechanism underlying the increased oleanolic acid content during fermentation require further investigation. In the negative ion mode, 63 types of lipids, 43 types of organic acids and their derivatives, 16 types of organic heterocyclic compounds, 12 types of nucleoside and nucleotide analogs, 12 types of benzene ring compounds, 11 types of organic oxidation compounds, and 5 types of phenylpropanoids and polyketones were identified.

**Figure 6:**
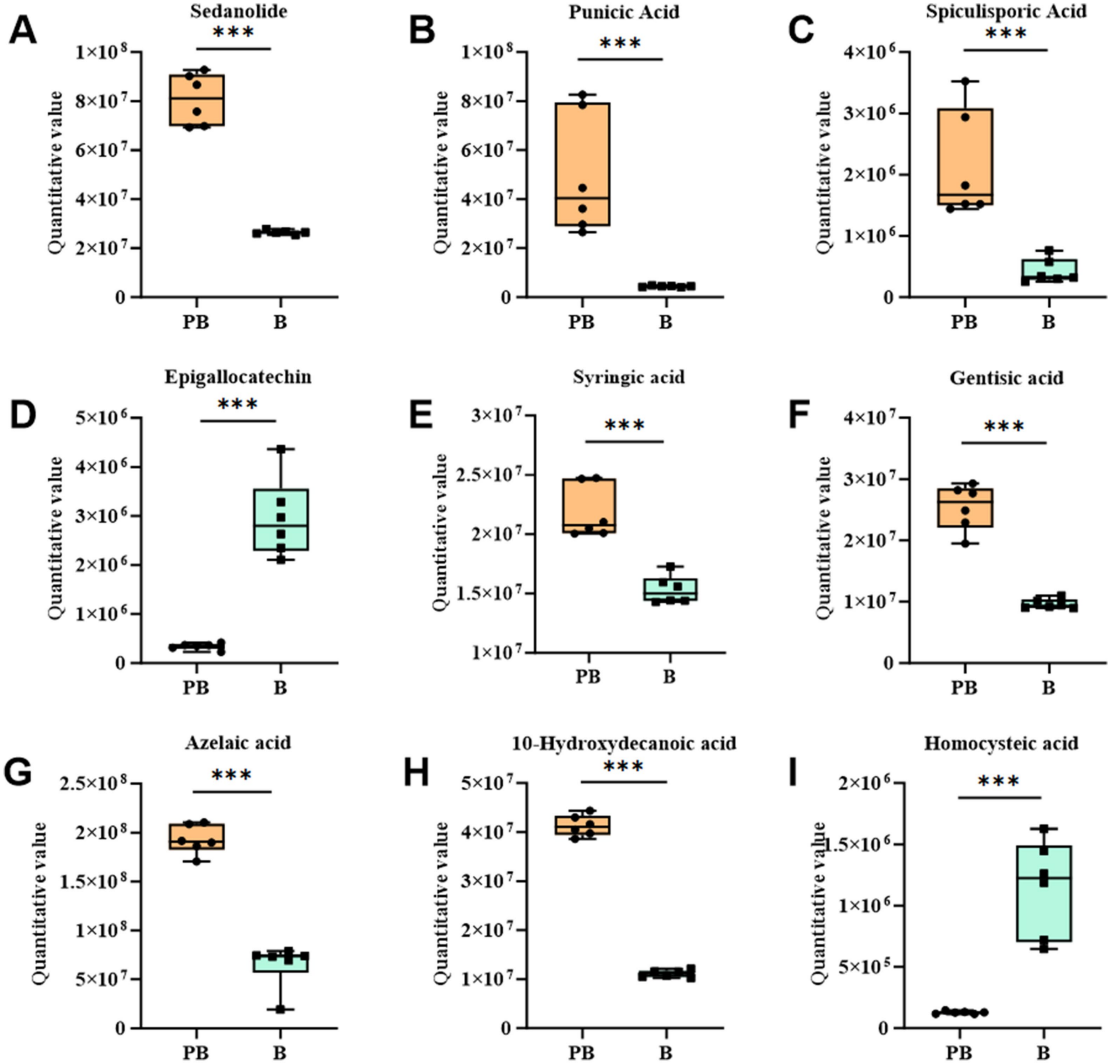
Boxplot changes in differential metabolites of antioxidant action. **(A)** Sedanolide, **(B)** punicic acid, **(C)** gamma-butenolide, **(D)** epigallocatechin, **(E)** syringic acid, **(F)** gentisic acid, **(G)** azelaic acid, **(H)** 10-hydroxydecanoic acid, and **(I)** homocysteine.

Metabolites associated with antioxidant activity, including sedanolide, punicic acid, epigallocatechin and homocysteine, were identified in the fermentation broth of the *P. cocos* group fermented by *L. reuteri* HM-R28 (Figures 6A–I). These metabolites are effective antioxidants that enhance cellular and tissue protection. In this study, the substantial increase in the contents of sedanolide and punicic acid (Figures 6A,B) may have significantly increased hydroxyl radical scavenging rate observed in the *P. cocos* group fermented with strain *L. reuteri* HM-R28 compared with that of the supernatant of strain *L. reuteri* HM-R28. These findings indicate that the synergistic fermentation of *P. cocos* and *Lactobacillus* from breast milk can enhance the antioxidant effect of strain *L. reuteri* HM-R28. Epigallocatechin gallate, a flavonoid-like compound, protects the biofilm from free radical-induced oxidized damage, although the stability is poor (36). Lactic acid bacteria can significantly enhance the biotransformation and metabolism of catechin and catechin derivatives (37). This may also contribute to the observed decline of the epigallocatechin gallate content in the female *L. reuteri* HM-R28 in this study (Figure 6D). Furthermore, homocysteine undergoes auto-oxidization generating reactive oxygen species that cause cellular causing damage (38), particularly affecting human cardiovascular and nervous system health negatively. However, homocysteine levels were significantly reduced during co-fermentation (Figure 6I), thereby reducing the risk of disease. In this study, the content of most metabolites with antioxidant effects in strain *L. reuteri* HM-R28 fermented *Poria* group showed a significant increase. Additionally, antioxidant assays showed a significant increase in antioxidant capacity following fermentation with *L. reuteri* HM-R28. Moreover, foods rich in antioxidants generally promote human health and aid in disease prevention (39). Therefore, the synergistic fermentation of *L. reuteri* HM-R28 and *P. cocos* can develop functional, healthy food with an antioxidant effect.

**Figure 7:**
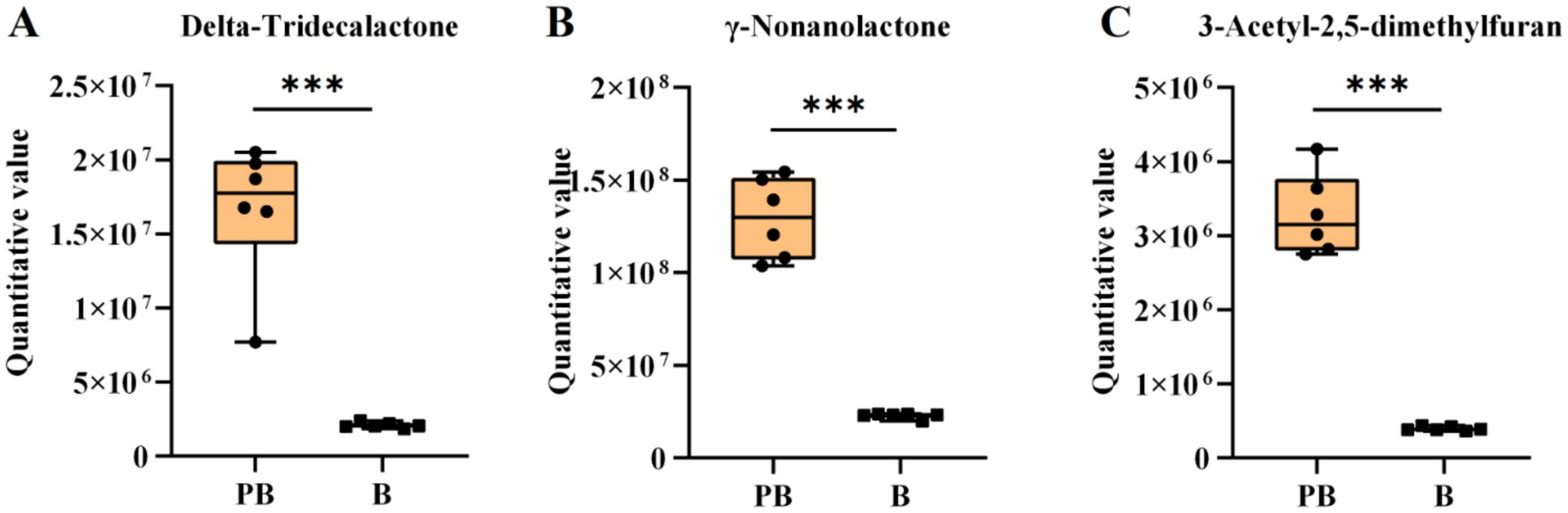
Boxplot variation of the volatile compound. **(A)** Delta-tridecanolactone, **(B)** *γ*-nonanolide, and **(C)** 3-acetyl-2,5-dimethylfuran.

*Poria* polysaccharides alone can stimulate the growth of *Lactobacillus* and *Bifidobacterium* (40). In this study, the contents of raffinose and other oligosaccharides in the fermentation medium of strain *L. reuteri* HM-R28 increased, while that of oligosaccharides in the fermentation medium of strain *L. reuteri* HM-R28 was very low. This suggests that strain *L. reuteri* HM-R28 promotes its growth and proliferation by converting pachyman into various oligosaccharides (Figures 7A–C). During the co-fermentation of *Saccharomyces cerevisiae* with *S. cerevisiae* and *Hanseniaspora uvarum* Yun268 respectively, the aroma of cider and wine is enhanced by adjusting the polyphenol and ethyl ester content thereby reducing sourness and bitterness (31, 41). The production of these aromatic compounds not only enhances the flavor and taste of *P. cocos* but also improves its health benefits, which can help diversify *P. cocos* products to meet the needs of various consumers.

### 3.7 KEGG pathway enrichment analysis

**Figure 8:**
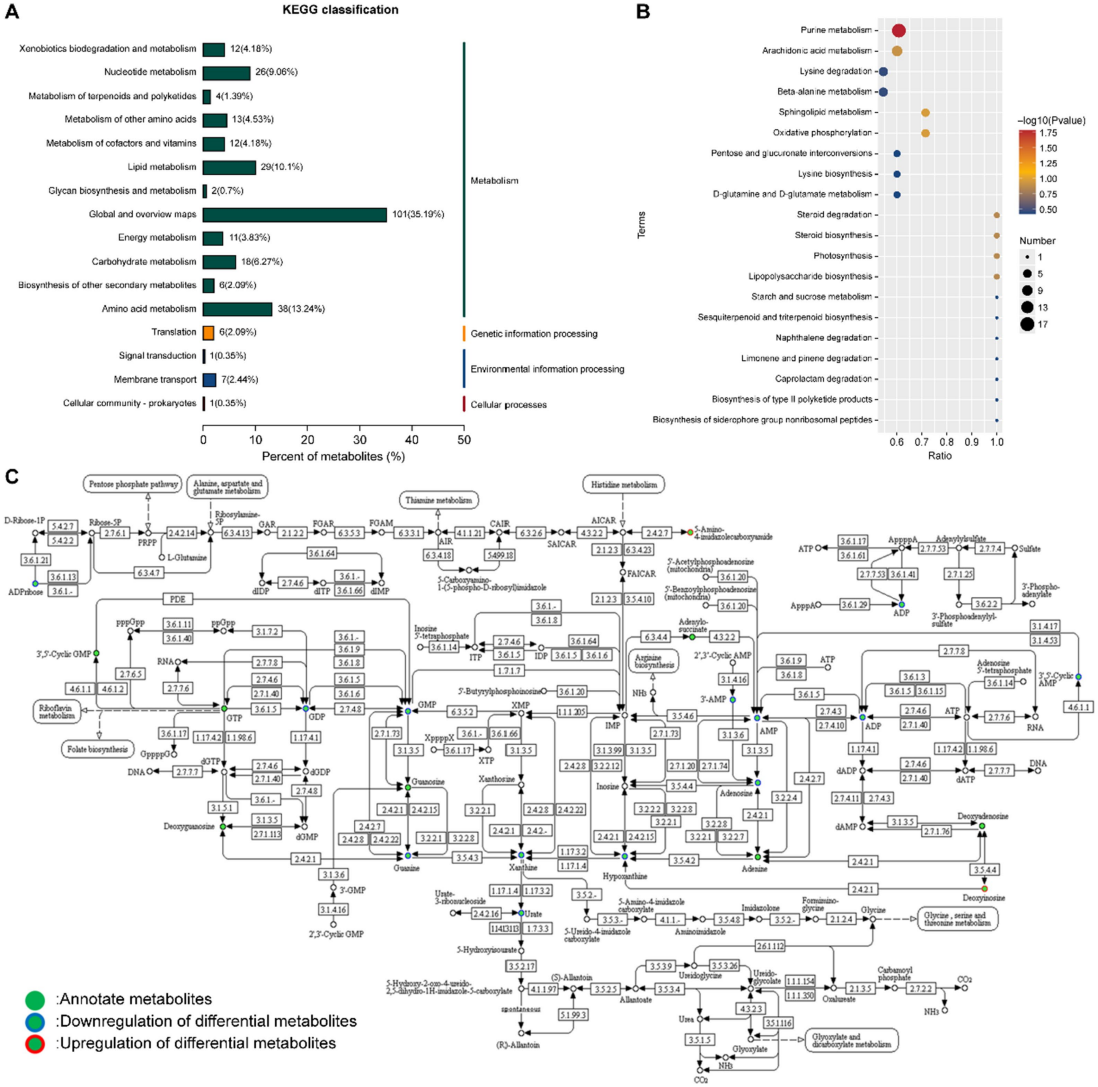
KEGG enrichment analysis of differential metabolites. **(A)** Classification analysis list and **(B)** enrichment result map. X/Y represents the coordinates, and the larger value indicates a higher concentration of metabolites along the path. Point color denotes the *p*-value of the super geometric test; smaller values indicate greater statistical significance and higher test reliability. Point size reflects the number of metabolites associated with each corresponding pathway, with larger points indicating greater differences in the pathway. **(C)** Purine metabolic pathway.

To further elucidate the relevant metabolic channels, the KEGG pathway database was used to analyze the difference in metabolites. The results showed that the strain *L. reuteri* HM-R28 fermented Poria group and strain *L. reuteri* HM-R28 group of different metabolites participated in 41 metabolic channels (Figure 8A). The effect of *L. reuteri* HM-R28 derived from breast milk on the metabolic pathways of *P. cocos* before and after fermentation was analyzed based on the *p*-value and influence value of each metabolic pathway (Figure 8B). Key metabolic pathways identified included purine, arachidonic acid metabolism, lysine degradation, *β*-alanine, and sphingolipid metabolism, in which the number of metabolites associated with purine metabolism, arachidonic acid metabolism, pyrimidine metabolism, and histidine metabolism increased significantly, which may be a key pathway for the synergistic effect of *L. reuteri* HM-R28 and *P. cocos in vivo*.

**Figure 9:**
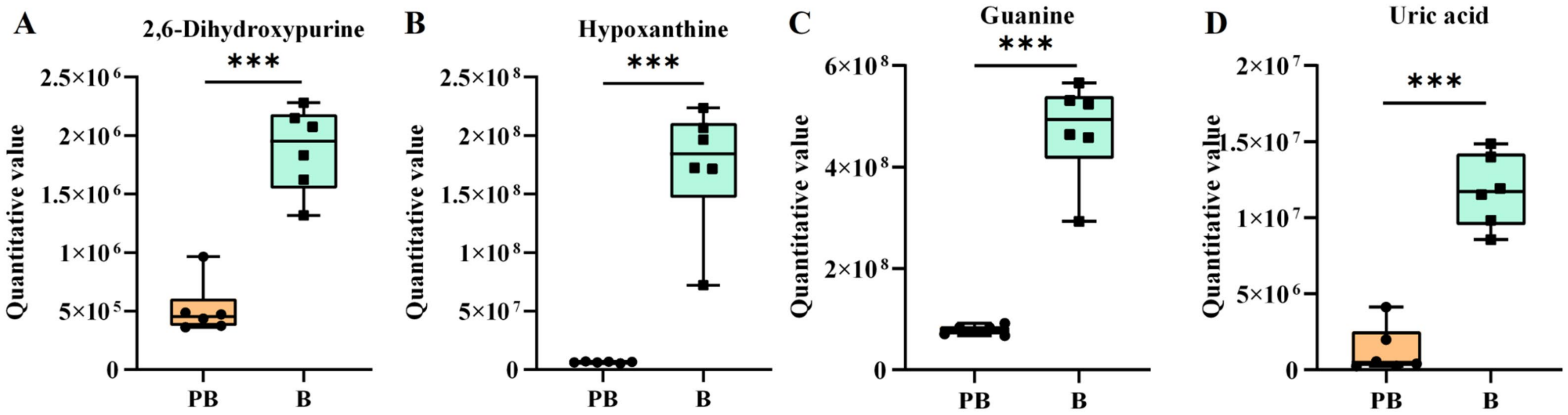
Differential metabolites involved in purine metabolism. **(A)** Xanthine, **(B)** hypoxanthine, **(C)** guanine, and **(D)** uric acid. *** indicates *p* < 0.001. B refers to the supernatant of the *L. reuteri* HM-R28 culture sample. PB represents the supernatant of the *Poria cocos* and strain *L. reuteri* HM-R28 co-fermentation sample.

The metabolic alterations in the fermentation broth of *P. cocos* are regulated by multiple substances and reactions. In this study, the purine metabolic pathway was significantly different (*p* = 0.0165), with 17 different metabolites identified in this pathway (Figure 8C). Key precursors of uric acid synthesis, including guanine, xanthine, hypoxanthine, and guanylic acid, were significantly reduced (Figures 9A–D), which may explain the observed decrease in uric acid content. While this study presents *in vitro* evidence demonstrating a reduction in purine metabolites following fermentation of *P. cocos* with *L. reuteri* HM-R28, the potential suitability of this product for patients with hyperuricemia is further supported by *in vivo* findings. For instance, a recent study (42) reported that a *Gardenia jasminoides–P. cocos* extract (GPE) significantly lowered serum uric acid levels in hyperuricemic rats by inhibiting xanthine oxidase activity and mitigating oxidative stress and inflammation. GPE treatment also improved renal function indicators (such as BUN and creatinine) and decreased pro-inflammatory cytokines (including TNF-*α* and IL-6), offering preclinical validation of the anti-hyperuricemic properties of *Poria*-based formulations. Although our work is limited to in vitro metabolic analysis, these in vivo results provide supporting evidence for the potential dietary application of *P. cocos* fermented with *L. reuteri* HM-R28 as a low-purine functional food for individuals with hyperuricemia.

## 4 Conclusion

The antioxidant activity and metabolomic profile of *L. reuteri* HM-R28 derived from breast milk were investigated after the fermentation of *P. cocos*. The results showed that strain *L. reuteri* HM-R28 may promote its own proliferation by converting *P. cocos* polysaccharides into oligosaccharides. Based on non-targeted metabolomics and KEGG pathway enrichment analysis, differential metabolites in the fermentation broth were significantly enriched in the purine metabolism pathway. Further integration with MetaCyc database data allowed the reconstruction of metabolic networks, enabling the prediction of metabolic pathway maps for characteristic differential metabolites. Though our findings highlighted advantages of *L. reuteri* HM-R28 derived from breast milk, further quantitative validation through targeted metabolomics, as well as confirmation of the bioactivity of fermentation products via cell culture and animal models are required. Additionally, a more comprehensive investigation into the specific transformation mechanisms of bioactive compounds during fermentation is warranted. Nonetheless, our findings provide a theoretical basis for the future development of functional health foods with antioxidant properties and low purine content.

## Data Availability

The original contributions presented in the study are included in the article/supplementary material, further inquiries can be directed to the corresponding authors.
